# Structure and Substrate Specificity of Human Short-Chain Acyl-CoA Dehydrogenase and Insights into Pathogenicity of Disease-Associated Mutations

**DOI:** 10.3390/ijms27062657

**Published:** 2026-03-14

**Authors:** Fang Bai, Xinru Li, Kaide Ju, Xijiang Pan, Ye Jin, Zhijing You, Lili Zhang, Zhaoxia Liu, Shuyang Zhang, Xiaodong Luan

**Affiliations:** 1 Department of Cardiology, Peking Union Medical College Hospital, Chinese Academy of Medical Sciences & Peking Union Medical College, Beijing 100730, China; 2 Center for Drug Research and Evaluation, Institute of Clinical Medicine, Peking Union Medical College Hospital, Chinese Academy of Medical Sciences & Peking Union Medical College, Beijing 100730, China; 3 School of Medicine, Tsinghua University, Beijing 100084, China; 4 Department of Rare Diseases, Peking Union Medical College Hospital, Chinese Academy of Medical Sciences & Peking Union Medical College, Beijing 100730, China

**Keywords:** cryo-EM, SCAD, gene mutation, mechanisms of disease, SCADD

## Abstract

Short-chain acyl-CoA dehydrogenase (SCAD) is a critical enzyme in mitochondrial fatty acid β-oxidation, catalyzing the initial dehydrogenation of short-chain acyl-CoAs. Mutations in the *ACADS* gene cause SCAD deficiency (SCADD), a disorder with remarkably heterogeneous clinical presentation. However, the molecular mechanisms underlying substrate specificity and the pathogenicity of most *ACADS* variants remain poorly understood. Here, we present high-resolution cryo-EM structures of human SCAD in complex with its physiological substrate butyryl-CoA (C_4_) and the longer substrate hexanoyl-CoA (C_6_). The butyryl-CoA-bound structure at 2.1 Å resolution details a pre-catalytic geometry ideal for hydride transfer, with Glu392 positioned as the catalytic base. We systematically characterized nineteen disease-associated mutations, which we classify into three functional categories: those disrupting FAD binding, those impairing substrate binding, and those compromising protein folding and stability. In addition, using the W177R mutant as a representative model, we demonstrate that folding-defective mutations provoke protein aggregation, leading to proteotoxicity, oxidative stress, and apoptosis, revealing a pathogenic mechanism beyond mere catalytic loss. In brief, our integrated findings elucidate the structural determinants of substrate specificity and catalytic mechanism in SCAD, and provide mechanistic insights into the functional impairments caused by mutations linked to SCADD.

## 1. Introduction

Short-chain acyl-CoA dehydrogenase (SCAD) is a pivotal mitochondrial flavoenzyme that catalyzes the α, β-dehydrogenation of short-chain acyl-CoA esters, the initial step in the mitochondrial fatty acid β-oxidation spiral [1,2,3]. As a member of the acyl-CoA dehydrogenase (ACAD) family, SCAD exhibits distinct substrate specificity for four- and six-carbon acyl chains (butyryl-CoA and hexanoyl-CoA), playing an indispensable role in cellular energy homeostasis and lipid metabolism [4,5]. The ACAD family includes enzymes with varying chain-length preferences: very long-, long-, medium-, and short-chain specific dehydrogenases (VLCAD, LCAD, MCAD, and SCAD), which are dedicated to fatty acid β-oxidation, as well as isovaleryl-CoA dehydrogenase (IVD) and short/branched-chain acyl-CoA dehydrogenase (SBCAD), which function in amino acid catabolism [6,7,8]. Although these enzymes share a common evolutionary origin and a conserved catalytic mechanism, each is characterized by a unique substrate profile critical for metabolic channeling [9]. SCAD ensures the efficient breakdown of short-chain fatty acids, thereby maintaining the balance between lipid catabolism and energy production.

Deficiency in SCAD (SCADD; OMIM #201470) is an autosomal recessive inborn error of metabolism caused by mutations in the *ACADS* gene [10,11,12]. The clinical presentation of SCADD is highly heterogeneous, ranging from severe neonatal metabolic decompensation (including hypoglycemia, metabolic acidosis, and lethargy) to milder, later-onset symptoms such as developmental delay, muscular hypotonia, and seizures, or even an asymptomatic course [5,10,13]. Biochemically, the condition is characterized by elevated levels of C_4_-carnitine in blood and increased excretion of ethylmalonic acid (EMA) in urine [13,14]. To date, over 80 pathogenic variants in *ACADS* have been reported, including missense, nonsense, and splice-site mutations [5]. These genetic alterations are known to affect enzyme folding, FAD cofactor binding, and catalytic activity to varying degrees [15,16]. Nevertheless, the precise molecular mechanisms by which specific mutations lead to loss of function and contribute to the disease spectrum remain largely uncharacterized, underscoring the need for integrated structural and functional studies.

Previous structural studies have provided foundational insights into SCAD’s architecture [17,18]. The reported three-dimensional structure of human SCAD, determined in a CoA-persulfide-bound form, revealed a homotetrameric quaternary assembly with each monomer binding one FAD prosthetic group and identifying Glu392 as the catalytic base [17]. However, the absence of an acyl moiety in the substrate-binding pocket in this structure has left critical questions unanswered regarding the precise determinants of substrate specificity and the complete catalytic machinery. A high-resolution structure of SCAD in complex with its native short-chain acyl-CoA substrate is essential to fully elucidate the structural basis of its enzymatic activity and chain-length selectivity.

In this study, we employed single-particle cryo-electron microscopy (cryo-EM) to determine the high-resolution structures of human SCAD in complex with its primary substrate, butyryl-CoA, and the longer substrate, hexanoyl-CoA. These structures reveal the precise geometry of the substrate-binding pocket and illuminate the molecular basis for substrate specificity and catalytic efficiency. Moreover, we systematically characterized 19 disease-associated SCAD mutation drawn from published reports [8,19,20], or the ClinVar database, comparing their biochemical properties with wild-type enzyme. Through systematic mutagenesis and biochemical analyses guided by our structural data, we delineate the mechanistic consequences of disease-associated mutations, classifying them into distinct functional categories. As a representative case, we demonstrate that the W177R folding-destabilizing mutation promotes protein aggregation. Importantly, this aggregation triggers a downstream pathogenic cascade involving oxidative stress and apoptosis, establishing a direct mechanistic link between protein misfolding and cellular toxicity. Our findings provide a comprehensive structural framework for understanding SCAD function and the pathophysiology of SCADD, offering insights that may inform future therapeutic strategies.

## 2. Results

### 2.1. Overall Architecture of SCAD

**Figure 1:**
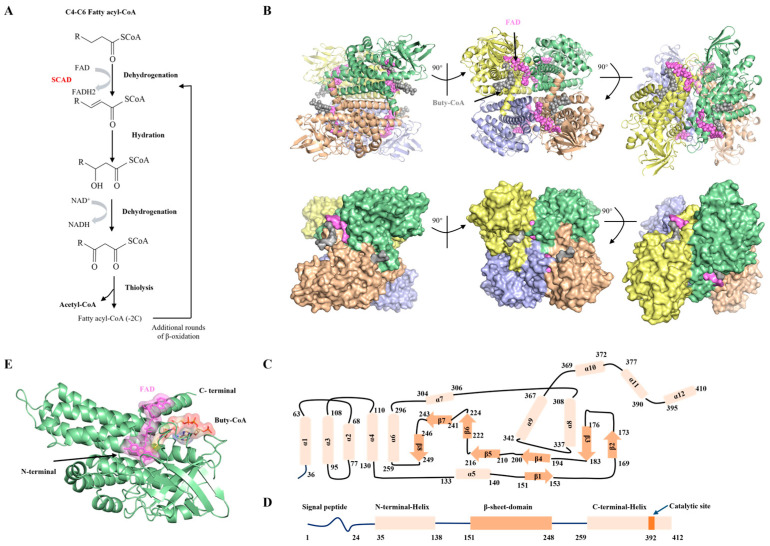
Overall structure of SCAD in complex with butyryl-CoA in different orientations. (**A**) SCAD catalyzes the dehydrogenation of short acyl-CoA thioester to the corresponding trans-2,3-enoyl-CoA, as part of mitochondrial β-oxidation. (**B**) Different views of the model of SCAD-butyryl complex are shown in cartoon (upper) and surface representation (lower). Monomers in tetramer were colored distinguishingly. (**C**,**D**) Schematic diagram of secondary structure of SCAD monomer. α-Helices are labeled as light orange cylinders, and the β-sheets are marked as orange arrows. (**E**) Representation of SCAD monomer structure with butyryl-CoA substrate and cofactor FAD (purple).

Mitochondrial fatty acid β-oxidation is central to cellular energy homeostasis, supplying approximately 80% of the ATP required for cardiac, skeletal muscle, and hepatic function [4]. This pathway consists of four sequential enzymatic steps—dehydrogenation, hydration, a second dehydrogenation, and thiolysis (Figure 1A), where each cycle shortening the acyl chain by two carbons to generate acetyl-CoA, reducing equivalents, and a shortened fatty acyl-CoA [21]. Among the enzymes involved, SCAD catalyzes the initial dehydrogenation step within the mitochondrial matrix, demonstrating highest catalytic activity toward butyryl-CoA (C_4_) and hexanoyl-CoA (C_6_) [5].

To elucidate the structural basis of substrate recognition, recombinant human SCAD was expressed in *E. coli* BL21(DE3) and purified to high homogeneity, as confirmed by size-exclusion chromatography and SDS-PAGE (Figure S1). SCAD is a member of the ACAD flavoprotein family and requires FAD as an essential cofactor. The purified protein exhibited a characteristic yellow color with a spectral absorbance maximum at 450 nm, consistent with FAD binding (Figure S1).

We determined the cryo-EM structure of SCAD in complex with butyryl-CoA at a global resolution of 2.1 Å (Figure S4), with detailed cryo-EM statistics provided in Supplementary Table S2. The high resolution is further evidenced by the clear density of aromatic side chains (Phe143 and Tyr391), which exhibit characteristic ring features. Furthermore, we observed clear density for butyryl-CoA in the substrate binding pocket (Figures S4 and S6), enabling atomic-level analysis of substrate binding. The overall quaternary structure of SCAD is a tetramer arranged as a dimer of dimers (Figure 1B), which was corroborated by static light scattering (SLS) showing a molecular mass of approximately 172 kDa (Figure S1). Each monomer adopts a fold conserved among ACADs, comprising an N-terminal α-helical domain (helices 1–5), a central β-sheet domain (strands 1–8) and a C-terminal α-helical domain (helices 6–12), presented in Figure 1C,D. The active site, housing both the FAD cofactor and the fatty acyl-CoA substrate, is situated at the interface of these three domains (Figure 1E). This architecture supports its catalytic function by facilitating optimal orientation of the substrate for α, β-dehydrogenation.

### 2.2. Structural Insights into the FAD and Substrate-Binding Pocket of SCAD

**Figure 2:**
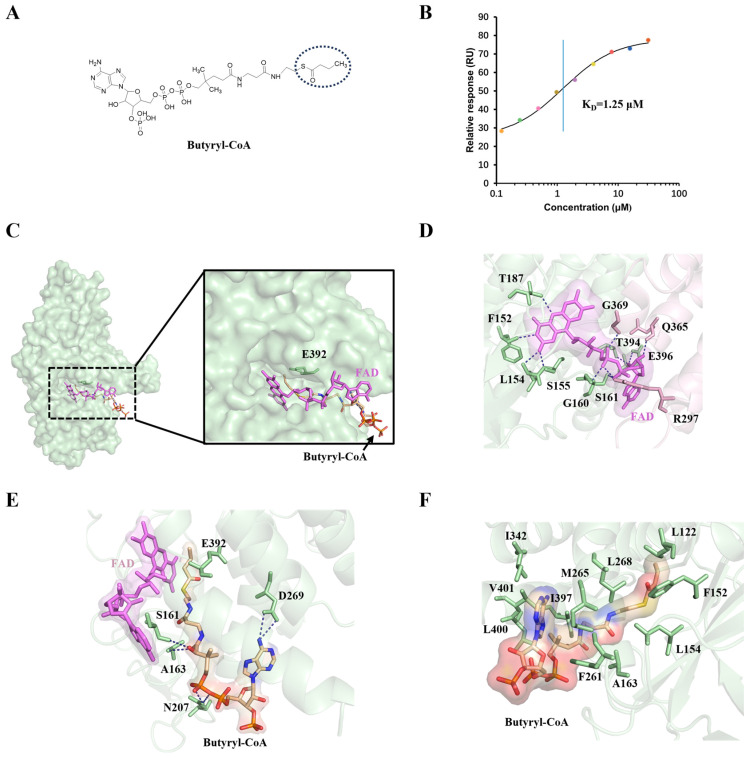
Structure of the active site of SCAD. (**A**) The chemical structures of butyryl-CoA. The acyl chain is highlighted by a circle. (**B**) SPR analysis of SCAD binding with butyryl-CoA. The solid line shows the global fit to the binding data (colored dots), yielding a K_D_ of 1.25 μM, which is indicated by the vertical blue line. (**C**) Monomer structure of the SCAD–butyryl-CoA complex. The deep-in views showing the locations of the FAD-binding site, the substrate-binding site, and active site (E392). (**D**) Hydrogen bond interactions between FAD and the surrounding residues in the SCAD–butyryl-CoA complex structure. Adjacent monomers were marked with different colors, and the FAD is shown in purple. (**E**) Residues that participate in hydrogen bonding interactions with butyryl are shown in green. Hydrogen bonds are represented by blue dashed lines. (**F**) The specific amino acid residues contributing to the formation of hydrophobic pockets around butyryl-CoA are displayed in detail. The residues in the hydrophobic pocket are marked in green.

SCAD demonstrates maximal catalytic activity toward butyryl-CoA, facilitating the conversion of acyl-CoA to 2-enoyl-CoA. This reaction is initiated by the abstraction of the pro-R α-proton from the thioester substrate, followed by hydride transfer from the pro-R β-carbon to the N_5_ atom of the isoalloxazine ring in enzyme-bound FAD [1,22]. To elucidate the structural determinants of substrate preference and catalytic mechanism, we determined the cryo-EM structure of SCAD in complex with butyryl-CoA and conducted complementary biochemical assays. Binding affinity measurements confirmed a strong interaction between SCAD and butyryl-CoA, with a dissociation constant (K_D_) of 1.25 μM (Figure 2B). Consistent with this, enzymatic activity assays indicated high catalytic efficiency with this substrate (Figure S2).

A well-defined density for FAD was observed in the active site, non-covalently bound and retained throughout purification, indicating stable incorporation of the cofactor. In the substrate-bound structure, the butyryl-CoA molecule is clearly resolved in the electron density map (Figures S4 and S6). The active site residue Glu392, which serves as the catalytic base, is positioned at the interface between the FAD-binding and substrate-binding domains, adjacent to the C_α_–C_β_ bond of the substrate (Figure 2C). The FAD cofactor is situated at the dimer interface, with its adenosine moiety engaging in interactions with a neighboring subunit within the functional dimer. Specifically, residues F152, L154, S155, and T187 from one monomer form hydrogen bonds with the isoalloxazine ring of FAD. Additional interactions with the ribityl and pyrophosphate groups are mediated by G160, S161, T364, and E396. Notably, R297, Q365, and G369 from the adjacent monomer also contribute to FAD binding through hydrogen bonds (Figure 2D), highlighting the critical role of the dimeric assembly in stabilizing the FAD-binding site.

The substrate-binding pocket is positioned opposite the isoalloxazine ring of FAD. Six hydrogen bonds are observed between protein residues and butyryl-CoA (Figure 2E). Among these, D269 interacts with the pantetheine arm of CoA, while S161, A163, and N207 engage with the acyl chain of butyryl-CoA. Disruption of these interactions is expected to impair substrate binding and catalysis. The acyl chain is positioned in proximity to the N_5_ atom of FAD, consistent with its role in α, β-dehydrogenation. Furthermore, the hydrophobic tunnel lining the substrate-binding cavity is composed of multiple nonpolar residues (Figure 2F), facilitating the optimal positioning of the aliphatic chain. Functional validation via mutagenesis revealed that the E392A mutant nearly abolishes enzymatic activity compared to wild-type SCAD (Figure S2), confirming the essential role of Glu392 as the catalytic base responsible for α-proton abstraction. Collectively, these structural features ensure precise substrate orientation and polarization, facilitating efficient catalysis within the SCAD active site.

### 2.3. Structural Basis for the Substrate Selectivity of SCAD

**Figure 3:**
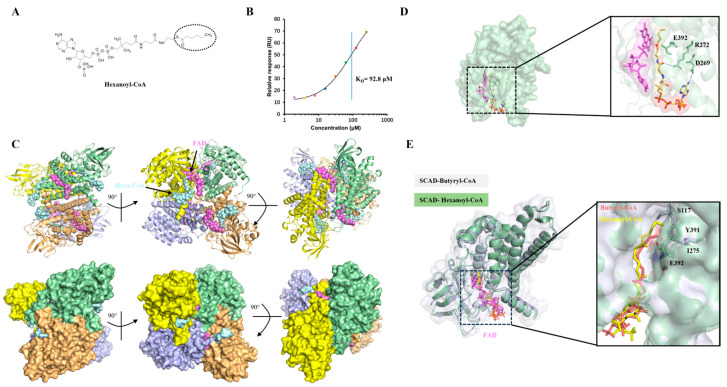
Structure of SCAD with hexanoyl-CoA. (**A**) The chemical structures of hexanoyl-CoA. The acyl chain is highlighted by a circle. (**B**) SPR analysis of SCAD binding with hexanoyl-CoA. The solid line shows the global fit to the binding data (colored dots), yielding a K_D_ of 92.8 μM, which is indicated by the vertical blue line. (**C**) Different views of the model of SCAD-hexanoyl-CoA complex are shown in cartoon (upper) and surface representation (lower). The four monomers of SCAD are presented in different colors. (**D**) Monomer structure of the SCAD–hexanoyl-CoA complex. The deep-in views showing the interaction details between SCAD and hexanoyl-CoA. Hydrogen bonds are represented by blue dashed lines. (**E**) Structural superposition of the SCAD-Butyryl-CoA (gray) and SCAD-Hexanoyl-CoA (green) complexes. The inset shows a close-up view of the substrate-binding pocket. Hexanoyl-CoA and Butyryl-CoA are displayed as yellow and orange sticks, respectively.

Substrate chain-length specificity is a fundamental characteristic within the ACAD family. To investigate the structural determinants underlying SCAD’s preference for short-chain acyl-CoAs, we sought to resolve the structure of SCAD in complex with hexanoyl-CoA (C_6_), a substrate longer than butyryl-CoA (C_4_) (Figure 3A). First, we quantified the binding affinity of SCAD for hexanoyl-CoA using SPR. The results indicated a significantly weaker interaction, with a K_D_ value of 92.8 μM (Figure 3B), compared to that for butyryl-CoA. To facilitate structural analysis, the SCAD–hexanoyl-CoA complex was formed using elevated substrate concentrations and subjected to cryo-EM. The cryo-EM structure was determined at a high resolution of 2.17 Å. Notably, the map quality allowed for the visualization of characteristic side-chain features, such as the density holes in aromatic rings (Phe341 and Tyr391). The well-resolved density map also allowed for the clear identification of the Hexanoyl-CoA ligand, enabling a detailed analysis of its binding mode (Figures S5 and S6). Structural analysis revealed that hexanoyl-CoA engages hydrogen bonds primarily with residues R272 and D269 (Figure 3D). The reduced number of polar interactions, relative to the butyryl-CoA complex, likely contributes to the lower binding affinity observed for hexanoyl-CoA.

Notably, the hexanoyl moiety adopts a bent conformation, as the side chains of S117, I275, and Y391 collectively form a steric barrier that restricts the extension of longer acyl chains (Figure 3E). This conformational adjustment may misalign the C_α_–C_β_ bond of the substrate relative to the catalytic base Glu392, compromising the optimal geometry required for pro-R α-proton abstraction. Consequently, this suboptimal positioning is likely a key factor reducing catalytic efficiency with longer-chain substrates. We further evaluated the binding of octanoyl-CoA (C_8_) to SCAD. As anticipated, the affinity was even weaker (K_D_ = 200 μM), consistent with the limited volume and length of the substrate-binding tunnel (Figure S3). These results indicate that SCAD’s active site is structurally constrained to accommodate shorter acyl chains. Binding of longer substrates necessitates conformational rearrangements that perturb the precise geometry of the catalytic center, thereby reducing both binding affinity and reaction rate.

Collectively, our structural and biochemical data delineate a dual mechanism governing substrate specificity in SCAD. For shorter-chain substrates like butyryl-CoA, the active site provides an optimal geometric and chemical environment for high-affinity binding and catalytically competent positioning. In contrast, longer acyl chains necessitate suboptimal binding modes characterized by reduced hydrogen bonding and, critically, a distorted alignment of the scissile bond relative to the catalytic base and the FAD cofactor. This “geometric frustration” model not only explains the diminished activity of SCAD toward longer-chain substrates, but also provides a critical structural framework for understanding how mutations that perturb the precise architecture of the active site, such as those found in patients with SCAD deficiency that can lead to pathological loss of function.

### 2.4. Functional Roles of Disease-Associated Mutations

Pathogenic variations in the *ACADS* gene (MIM*606885) encoding SCAD are associated with SCAD deficiency (SCADD), a disorder characterized by elevated urinary excretion of EMA, a metabolite derived from accumulated butyryl-CoA, as well as heterogeneous clinical manifestations such as developmental delay, seizures, and hypotonia [23,24]. The clinical presentation of SCADD varies widely, from asymptomatic individuals to severe cases, and is paralleled by heterogeneity in biochemical and mutational profiles [25]. To elucidate the mechanistic impact of disease-associated mutations, we introduced several known pathogenic and rare variants from ClinVar database into recombinant SCAD and assessed their effects on enzymatic activity, binding affinity, and protein stability.

**Figure 4:**
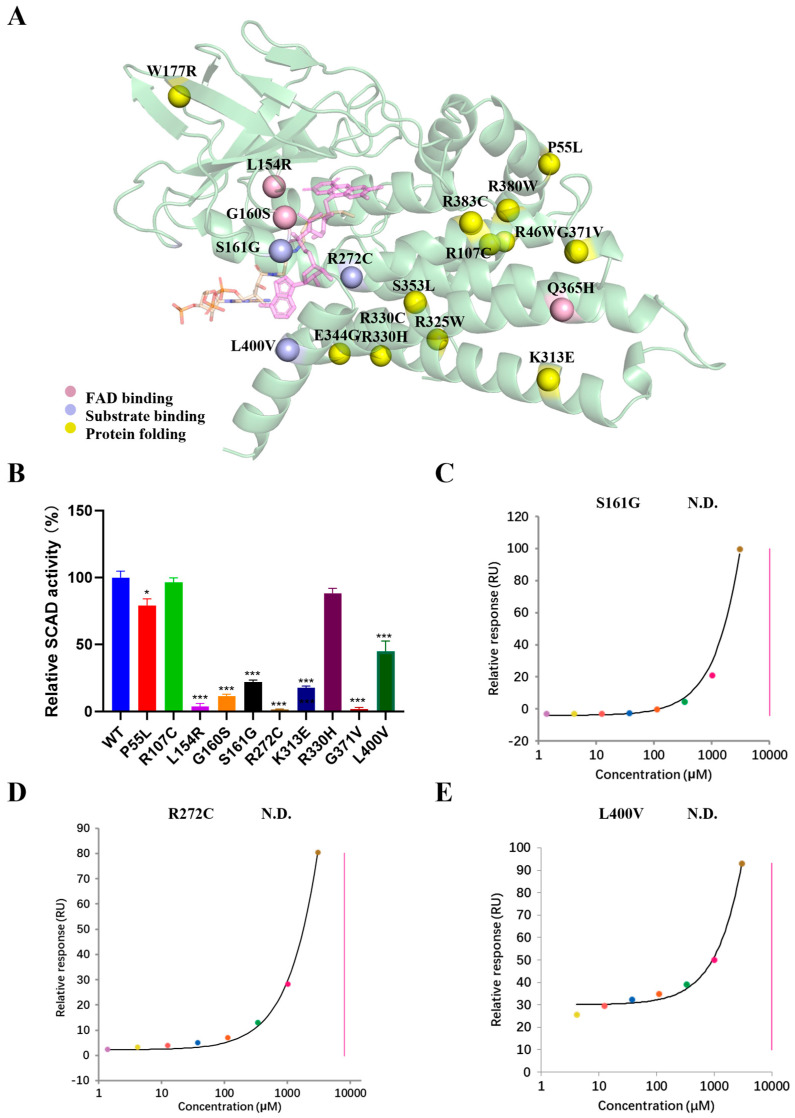
Analysis of the functional roles of the disease-associated mutations of SCAD. (**A**) Locations of the disease-associated mutations in the structure of SCAD. Mutations affecting the FAD binding, the substrate binding, and the protein folding and stability are displayed with pink, purple and yellow spheres, respectively. (**B**) Residual activity of the disease- associated SCAD mutants, shown as % of the wild-type SCAD activity using butyryl-CoA as substrate. The values are presented as the means ± SEM. (**C**–**E**): SPR analysis of S161G (**C**), R272C (**D**) and L400V (**E**) corresponding to the disease-associated SCAD mutants for butyryl-CoA was performed. The solid line represents the global fit to the binding data (colored dots). N.D.: Not determined, no valid K_D_ could be obtained from the fit. * *p* < 0.05, *** *p* < 0.001.

Among the mutations in the study, three are located within the FAD-binding domain and three within the substrate-binding domain (Figure 4A). Mutants L154R and G160S, both situated in the FAD-binding site, exhibited severely impaired enzymatic activity compared to the wild-type (Figure 4B). Structural analysis indicates that these substitutions disrupt critical interactions with FAD, compromising cofactor binding and catalytic function. Notably, the Q365H variant prevented tetramer formation during purification. This residue, located at the subunit interface, forms a hydrogen bond with the FAD moiety of the adjacent subunit, suggesting that the mutation perturbs dimerization and tetramer assembly, thereby destabilizing the quaternary structure. Several mutations within the substrate-binding domain including S161G, R272C and L400V, also significantly reduced enzymatic activity. SPR analyses confirmed that these variants substantially decrease binding affinity for butyryl-CoA (Figure 4C–E).

Interestingly, some mutations located outside the FAD or substrate-binding sites resulted in markedly reduced soluble expression, indicative of impaired folding and stability. These stability-defective variants segregated into three subgroups based on residual activity levels: K313E and G371V exhibited minimal residual activity despite being purified; P55L, R107C, and R330H retained moderate to high specific activity; and a third group—including R46W, W177R, R325W, R330C, E344G, S353L, R380W, and R383C—could not be isolated in sufficient quantities for enzymatic assays under standard conditions (Supplementary Table S1), consistent with severe folding defects. Collectively, based on our structural and functional data, disease-associated mutations in SCAD can be categorized into three phenotypic classes: (i) those that disrupt FAD binding (e.g., L154R, G160S), abolishing enzymatic activity; (ii) those that impair substrate binding (e.g., S161G, R272C, L400V), reducing catalytic efficiency; and (iii) those that compromise protein folding and stability, leading to low expression and loss of function.

In summary, our integrated structural and functional analysis allows for a mechanistic classification of SCADD-associated mutations into three distinct pathophysiological categories. Notably, the category (iii)—particularly severe folding mutants such as W177R, not only diminishes functional protein levels but may also induce proteotoxicity via aggregation, a mechanism we further investigated in subsequent cellular models.

### 2.5. The W177R Mutation Triggers Protein Misfolding and Aggregation in Cells

**Figure 5:**
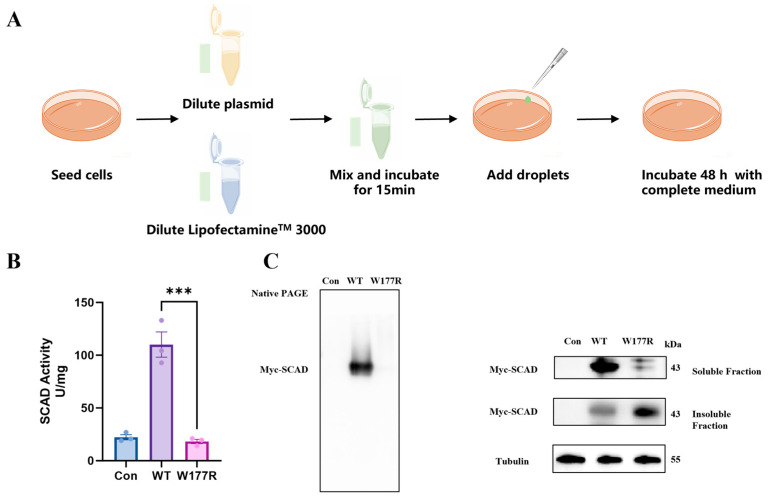
The W177R mutation reduces the formation of SCAD protein tetramers and promotes SCAD aggregation in 293T cells. (**A**) Plasmids carrying either the WT or W177R *ACADS* alleles were introduced into HEK293T cells via liposome-mediated transfection. (**B**) SCAD activity of cell lysates from 293T cells transfected with WT or W177R plasmid DNA. The results were presented as activity units/mg protein. (**C**) Immunoblot analysis of HEK293T cells transfected with WT or W177R plasmid DNA. Cellular lysates were fractionated into a soluble and an insoluble fraction using native lysis buffer. The soluble fraction was subjected to Native-PAGE analysis (left) and SDS–PAGE Western blotting (right), while the insoluble fraction (resuspended in equal volume) was analyzed by SDS-PAGE (right). Tubulin serves as the loading control for the soluble fraction. The experiments were repeated three times. All data were presented as mean ± SEM; *** *p* < 0.001.

To validate the hypothesis that folding-destabilizing mutations lead to loss of function via protein aggregation, we selected the W177R mutant—which exhibited virtually no soluble expression in recombinant form—for further mechanistic investigation in 293T cells. We transfected cells with c-Myc–tagged constructs encoding either WT or W177R SCAD and analyzed their expression and functional properties (Figure 5A). Enzymatic activity assays performed on total cell lysates 48 h post-transfection revealed that cells expressing WT SCAD showed a significant increase in activity, whereas those expressing W177R displayed activity levels comparable to empty vector controls (Figure 5B), indicating a severe functional impairment. To investigate the molecular basis of this defect, we separated cell lysates into soluble and insoluble components to assess protein assembly and solubility. Native-PAGE analysis of the soluble fraction showed a strong band corresponding to tetrameric WT SCAD, while the signal for W177R was markedly diminished (Figure 5C), suggesting defective quaternary structure formation. Consistently, SDS-PAGE analysis revealed that W177R SCAD was undetectable in the soluble fraction but was entirely recovered in the insoluble pellet (Figure 5C), confirming its aggregation-prone behavior in a cellular context. These data demonstrate. These data demonstrate that the W177R mutation disrupts normal protein folding, prevents tetramerization, and promotes aggregation into insoluble species, thereby abrogating enzymatic function.

### 2.6. Cells Expressing the W177R Mutant Protein Induced Oxidative Stress and Apoptosis

**Figure 6:**
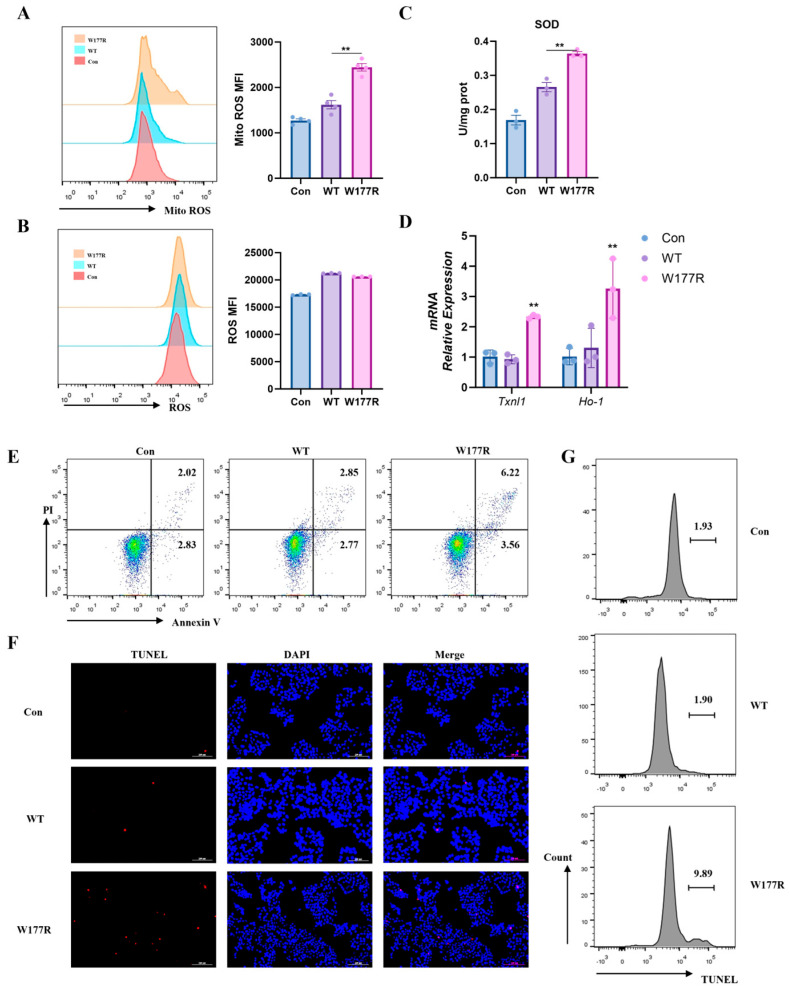
Cells expressing the W177R mutant protein triggered oxidative stress and apoptosis. (**A**) The level of Mito-ROS and ROS (**B**) in 293T cells expressing WT and W177R was analyzed by FACS (MFI: mean fluorescence intensity). (**C**) SOD activity of cell lysates from 293T cells transfected with WT or W177R plasmid DNA. (**D**) The mRNA level of *TXNL1* and *HO-1* in 293T cells transfected with WT or W177R plasmid DNA. (**E**) Flow cytometry analysis of cell death (Annexin V^+^ PI^+^) in 293T cells expressing WT and W177R. (**F**) Representative fluorescence images of 293T cells expressing WT or W177R after a TUNEL assay. Apoptotic cells are indicated by TUNEL staining (red), and nuclei are counterstained with DAPI (blue). Scale bar, 100 μm. (**G**) Flow cytometry analysis showing a TUNEL-positive cell population. The experiments were repeated three times. All data were presented as mean ± SEM; ** *p* < 0.01.

Accumulated misfolded proteins typically exert a toxic effect on cells, triggering oxidative stress. To further evaluate whether the mutation perturbed the mitochondria homeostasis, we assessed mitochondrial superoxide generation among different transduced cells, using a Mito-SOX probe. Unsurprisingly, mitochondrial superoxide levels were significantly elevated in cells carrying the W177R mutation (Figure 6A). However, no apparent increase in cytoplasm ROS levels was observed in cells harboring W177R mutant compared with WT SCAD (Figure 6B). These results suggest that the W177R mutant protein may activate the cellular antioxidant system in response to elevated mitochondrial ROS levels. As expected, the activity of the anti-oxidant enzyme superoxide dismutase (SOD) was upregulated in cells carrying W177R (Figure 6C). Correspondingly, the gene expression of anti-oxidant enzymes including *TXNL-1* and *HO-1* in cells transduced W177R was also upregulated (Figure 6D). These findings suggested that the W177R mutant could induce an increase in mitochondrial ROS, which in turn activated intracellular antioxidant enzymes to maintain cellular homeostasis.

The accumulation of ROS from mitochondria might lead to cell death via apoptosis [26,27]. We monitored the level of apoptosis in WT or W177R-expressing cells using the Annexin V-FITC/PI double-staining apoptosis assay (Figure 6E). Our result showed that cell apoptosis was significantly elevated under W177R overexpression compared with WT. In addition, the TUNEL assay also showed the presence of typical characteristics of apoptosis in the W177R group (Figure 6F,G). Therefore, these data indicate that the W177R mutation likely leads to protein misfolding and aggregation, which in turn triggers cellular oxidative stress and apoptosis in 293T cells.

## 3. Discussion

SCAD is a key enzyme in mitochondrial fatty acid β-oxidation, catalyzing the dehydrogenation of four- or six-carbon acyl-CoA substrates to form corresponding 2-enoyl-CoA products, supporting cellular energy production [5]. In this study, we integrated cryo-EM structures of substrate-bound SCAD with comprehensive biochemical and cellular assays, to elucidate the molecular basis of SCAD function and dysfunction. Our findings elucidate the structural determinants of substrate specificity and catalytic mechanism in SCAD, and establish a structure-based taxonomy of disease-causing mutations, linking their biophysical impacts to cellular pathology.

The overall architecture of SCAD is consistent with the conserved fold of the ACAD family [28]. SCAD functions as a homotetramer arranged as a dimer of dimers, with each monomer comprising three domains: an N-terminal α-helical domain, a central β-sheet domain, and a C-terminal α-helical domain. The FAD cofactor is bound at the interface of these three domains. Notably, the flavin ring also engages residues from the C-terminal domain of the adjacent subunit within the dimer, indicating that FAD not only participates in electron transfer but also plays a structural role in stabilizing the oligomeric assembly.

ACADs exhibit chain-length specificity, and SCAD is most active toward four-carbon acyl-CoAs. Our data confirm that SCAD dehydrogenates both butyryl-CoA and hexanoyl-CoA, albeit with a 10-fold higher binding affinity for butyryl-CoA (Figure 2B). Structural comparison with the previously reported SCAD structure (PDB ID: 8SGS) reveals a highly consistent overall architecture (Figure S7), while our high-resolution map provides clearer density for the ligand acyl chain. Structural analysis reveals that the substrate-binding pocket is relatively shallow and optimally shaped to accommodate short-chain acyl groups. In the butyryl-CoA-bound structure, the acyl moiety binds adjacent to the *re*-face of the FAD isoalloxazine ring, with Glu392 positioned ideally near the C_α_–C_β_ bond to act as the catalytic base. This configuration facilitates the stereospecific abstraction of the pro-R α-hydrogen and hydride transfer to FAD N_5_. In contrast, hexanoyl-CoA binds in a bent conformation, displacing the scissile bond away from Glu392 and resulting in suboptimal geometry for catalysis. This structural distortion underlies the reduced catalytic efficiency toward longer substrates. Our findings support a model wherein substrate specificity is governed not only by the volume of the binding pocket but also by the precise alignment of catalytic residues relative to the substrate.

SCADD is an autosomal recessive disorder caused by mutations in the *ACADS* gene and exhibits considerable clinical heterogeneity, ranging from severe metabolic decompensation to asymptomatic presentations [29]. We systematically characterized 19 disease-associated variants and categorized them into three functional classes: (i) those that disrupt FAD binding or tetramerization (e.g., L154R, G160S, Q365H); (ii) those that impair substrate binding and positioning (e.g., S161G, R272C, L400V); and (iii) those that compromise protein folding and stability, leading to reduced soluble expression (e.g., R46W, R383C, W177R). This classification directly links molecular lesions to biochemical outcomes, offering a structure-based framework for interpreting genotype-phenotype relationships in SCADD. Mutations that severely disrupt folding or cofactor binding likely underlie severe clinical presentations, whereas hypomorphic alleles that retain partial activity may manifest as milder or asymptomatic disease.

It is reported that the pathogenesis of SCADD is associated with abnormal protein aggregation and oxidative stress [11]. Beyond the loss of enzymatic function, we demonstrate that folding-defective mutations can actively confer proteotoxicity. Using W177R as an exemplar, we show that the mutant protein fails to adopt its native tetrameric state, instead forming insoluble aggregates. These aggregation phenomena in turn trigger mitochondrial oxidative stress, as evidenced by elevated superoxide levels. Consistent with this, we observed significant upregulation of antioxidant markers, including *TXNL*-1 and *HO-1*. Importantly, SOD, a key mitochondrial enzyme responsible for converting superoxide radicals to hydrogen peroxide and oxygen, and whose expression is induced under mitochondrial oxidative stress [30,31], was also markedly elevated. This pattern aligns with previous clinical observations of enhanced antioxidant responses in SCADD patients [25]. Furthermore, given that sustained high ROS levels can damage cellular macromolecules and compromise viability [23,32], we assessed apoptotic outcomes and confirmed that W177R expression significantly increased apoptosis (Figure 6E–G). Taken together, these findings support a complete pathogenic cascade: mutation-induced structural destabilization promotes protein aggregation, which triggers mitochondrial oxidative stress, leading to compensatory antioxidant activation and ultimately apoptosis.

In summary, our multi-scale investigation, integrating structural biology, biochemistry, and cellular assays, provides a comprehensive understanding of SCAD function and dysfunction. We have elucidated the structural basis of substrate specificity through cryo-EM structures, established a mechanistic classification of disease-associated mutations, and revealed how folding-defective variants like W177R initiate a pathogenic cascade from atomic-level instability to cellular apoptosis. These insights advance the mechanistic understanding of SCADD pathogenesis, and provide a structural framework for future therapeutic development targeting different classes of SCAD mutations.

## 4. Materials and Methods

### 4.1. Expression and Purification of Human Wild-Type and Mutant SCAD

The protein was expressed and purified as previously described [33]. Briefly, the expression plasmid pET28a-SCAD was transformed into *Escherichia coli* strain BL21(DE3). The cells were grown in Luria–Bertani (LB) medium at a temperature of 37 °C to an optical density (A600) of 0.6–0.8. To induce SCAD expression, isopropyl β-D-1-thiogalactopyranoside (IPTG) was added to a final concentration of 0.5 mM and grown at 16 °C for 16 h. The cells were then harvested and resuspended with a buffer containing 50 mM HEPES, pH 7.4, 300 mM NaCl, and 5% glycerol. Collected cells were then lysed using high-pressure machine and centrifugated at 12,000 rpm. After that, the supernatant loaded on a Ni-NTA (Qianchun Bio, Jiaxing, China) column for affinity chromatography and further purified by size exclusion chromatography using a Superdex 200 10/300 GL column (GE Healthcare, Chicago, IL, USA) in SEC buffer (25 mM HEPES, pH 7.4, 150 mM NaCl, and 20 mΜ FAD).

### 4.2. Cryo-EM Sample Preparation

The complex sample is obtained by incubating protein with substrates at 4 °C for 15 min. A total of 3 μL of the protein sample was applied to a glow discharged Quantifoil R1.2/1.3 300 mesh holey carbon grid. Grids were blotted for 4 s under 100% humidity and plunge-frozen in liquid ethane using a Vitrobot (Thermo Fisher Scientific, Pittsburgh, PA, USA).

### 4.3. Cryo-EM Data Collection and Processing

Data collection was acquired at a 300 kV Titan Krios transmission electron microscope (Thermo Fisher Scientific) equipped with a K2 electron detector and GIF BioQuantum energy filter (Gatan, Pleasanton, CA, USA) or Falcon4 detector and Selectris (Thermo Fisher Scientific). Raw images were recorded at magnification of 105,000× or 165,000×, which equals a physical pixel size of 0.82 Å per pixel or 0.73 Å per pixel and a nominal defocus range of 0.6–2.0 μm. For all cryo-EM initial image, motion correction and contrast transfer function (CTF) estimation were performed using cryoSPARC 4.7.1 with default settings. For each dataset, approximately 300 images were selected for Blob picking in cryoSPARC, and the resulting particles were sorted with 2D classification by structure features. These extracted particles were undergoing 2D classification to generate primary 3D models and further heterogeneous refinement. The subsequent local refinement was obtained using the mask generated from the non-uniform refinement. Following model building and further adjustments were performed using CooT 0.9.8.96. Finally, constructed models were validated in PHENIX 1.21.2.

### 4.4. Cell Culture and Transfection

HEK293T cells were purchased from Kebai (Cat#60439, Nanjing, China), and were cultured in DMEM supplemented with 10% FBS and 1% penicillin/streptomycin [34]. The *ACADS* cDNA was introduced into the mammalian expression vector pcDNA3.1-c-myc, and the resulting constructs carrying either the wild-type (WT) or W177R allele were transfected into HEK293 cells using Lipofectamine™ 3000 (Thermo Fisher Scientific, Pittsburgh, PA, USA). Cells were seeded at 1 × 10^6^ per well in 6-well plates and transfected with 5 µg plasmid after 24 h culturing. Then the cells were harvested 48 h post-transfection for analysis.

### 4.5. Enzyme Activity Assay

The activity of SCAD was assayed spectrophotometrically following the decrease in absorbance at 600 nm using phenazine methosulfate (PMS) and 2,6-dichlorophenolindophenol (DCPIP) as substitute intermediate and terminal electron acceptors, respectively (ε600 nm = 21 mM^−1^ cm^−1^) [35,36]. A standard assay mixture contained 20 mM phosphate buffer, pH 7.4, 100 μM DCPIP, 1.5 mM PMS, 30 μM EDTA, 20 μM FAD, and 100 μM butyryl-CoA. A total of 0.75 μg of purified protein or 20 μL cell lysis was added to initiate the reaction with a final volume of 200 μL, as described previously [23]. The reaction was monitored by measuring the decrease in DCPIP absorbance at 600 nm using a 96-well plate reader for 60 s at 37 °C. Specific enzyme activities were expressed as nmol of DCPIP reduced per min per mg of SCAD protein (nmol DCPIP·min^−1^·mg^−1^).

### 4.6. Western Blotting

Transfected cells were lysed on ice using a buffer containing 20 mM HEPES, 0.15 mM KCl, 1.5 mM MgCl_2_, 1 mM EDTA, 0.5% Triton X-100, and 10% glycerol, supplemented with protease and phosphatase inhibitors. The lysates were subjected to three freeze–thaw cycles and sonicated for 90 s. Soluble and insoluble fractions were separated by centrifugation at 17,000× *g* for 10 min at 4 °C. Both the supernatant (soluble fraction) and the pellet (insoluble fraction) were analyzed by Western blotting. Proteins were resolved on 10% SDS-PAGE or Native PAGE gels and transferred to PVDF membranes (Immobilon-P™, Millipore, Burlington, MA, USA, 0.45 μm). Membranes were blocked for 1 h in blocking buffer (Beyotime, Shanghai, China) and then incubated overnight at 4 °C with primary antibodies against c-Myc and tubulin. After washing with PBST (PBS containing 0.1% Tween-20), the membranes were incubated with HRP-conjugated secondary antibodies for 1 h at room temperature. Protein signals were detected using an ECL system (Amersham Bioscience, Buckinghamshire, UK).

### 4.7. Flow Cytometry Analysis on Apoptosis

Apoptosis in transfected 293T cells was assessed using an Annexin V-FITC Apoptosis Detection Kit (BD Pharmingen, San Diego, CA, USA). As previously described [37], cells were stained with Annexin V-FITC and propidium iodide (PI) for 15 min at 25 °C. Samples were analyzed immediately using a BD LSR Fortessa flow cytometer (BD Biosciences, San Jose, CA, USA), and data were processed with FlowJo_V10 software.

### 4.8. TdT-Mediated dUTP Nick-End Labeling (TUNEL)

Apoptotic cells were detected using a TdT-mediated dUTP Nick-End Labeling (TUNEL) assay kit (C1089, Beyotime, China) following the manufacturer’s protocol. In brief, transfected cells were fixed, permeabilized, and incubated with the TUNEL reaction mixture. After washing, nuclei were counterstained with DAPI. Fluorescence images were captured using a Nikon microscope (Tokyo, Japan).

### 4.9. RNA Extraction and RT-PCR Analysis

Total RNA from transfected 293T cells was extracted using the simple total RNA kits (Tiangen, Beijing, China). Then cDNA was synthesized with Hifair™ II 1st Strand cDNA Synthesis SuperMix for qPCR (Yeasen, Shanghai, China). Subsequently, SYBR^®^ Green Master Mix (Yeasen, Shanghai, China) and gene-specific primers were used for RT-PCR on QuantStudio 5 (Thermo Fisher Scientific, Pittsburgh, PA, USA), as described previously [37]. The primers used for RT-PCR were presented as following: *ACTB*-F: AGCGAGCATCCCCCAAAGTT; *ACTB*-R: GGGCACGAAGGCTCATCATT; *TXNL1*-F: CTCGCCGTGGTCAAGT; *TXNL1*-R: GGGTCATTTTCTAAGTGCTG; *HO-1*-F: GCCTACACCCGCTACCT; *HO-1*-R: CTCCTGCAACTCCTCAAAG. The fold change in mRNA expression of gene was standardized to *ACTB* using the ΔΔCt method.

### 4.10. SPR Assays

The binding affinities of SCAD and SCAD mutants for butyryl-CoA were performed by Biacore 8K Plus (Cytiva, Marlborough, MA, USA) at 25 °C. Proteins were immobilized on a CM5 sensor chip (Cytiva, Marlborough, MA, USA) via amine coupling. Serial dilutions of substrates in HEPS buffer were injected over the chip surface. Binding kinetics were evaluated using the Biacore Insight Evaluation Software 6.0.7.1750, and equilibrium dissociation constants (K_D_) were derived by fitting to a 1:1 binding model.

### 4.11. Statistical Analysis

All experiment data were presented as mean ± standard error of the mean (SEM). Statistical analyses were performed with One-way ANOVA followed by Dunnett’s test by GraphPad Prism 10.0 software. *p* < 0.05 was considered as a significant difference.

## Data Availability

The cryo-EM density maps have been deposited in the Electron Microscopy Data Bank (EMDB) under the accession codes EMD-66500 and EMD-66565, and the coordinates have been deposited in the Protein Data Bank (PDB) under the accession codes 9X3I and 9X4S. The original contributions presented in this study are included in the article/Supplementary Materials. Further inquiries can be directed to the corresponding author(s).

## Supplementary Materials

The supporting information can be downloaded at: https://www.mdpi.com/article/10.3390/ijms27062657/s1.

## Abbreviations

ACAD, acyl-CoA dehydrogenase; Cryo-EM, cryo-electron microscopy; EMA, ethylmalonic acid; FAD, flavin adenine dinucleotide; IVD, isovaleryl-CoA dehydrogenase; SBCAD, short/branched-chain acyl-CoA dehydrogenase; SCAD, short-chain acyl-CoA dehydrogenase; SCADD, SCAD deficiency.

## References

[1] Saenger A.K., Nguyen T.V., Vockley J., Stankovich M.T. (2005). Biochemical and electrochemical characterization of two variant human short-chain acyl-CoA dehydrogenases. Biochemistry.

[2] Pedersen C.B., Kølvraa S., Kølvraa A., Stenbroen V., Kjeldsen M., Ensenauer R., Tein I., Matern D., Rinaldo P., Vianey-Saban C. (2008). The ACADS gene variation spectrum in 114 patients with short-chain acyl-CoA dehydrogenase (SCAD) deficiency is dominated by missense variations leading to protein misfolding at the cellular level. Hum. Genet..

[3] Kim S.H., Park H.D., Sohn Y.B., Park S.W., Cho S.Y., Ji S., Kim S.J., Choi E.W., Kim C.H., Ko A.R. (2011). Mutations of ACADS gene associated with short-chain acyl-coenzyme A dehydrogenase deficiency. Ann. Clin. Lab. Sci..

[4] Faraji H., Ebrahim-Habibi A. (2024). Structural insights into the pathogenicity of point mutations in human acyl-CoA dehydrogenase homotetramers. J. Biol. Phys..

[5] Lisyová J., Chandoga J., Jungová P., Repiský M., Knapková M., Machková M., Dluholucký S., Behúlová D., Šaligová J., Potočňáková Ľ. (2018). An unusually high frequency of SCAD deficiency caused by two pathogenic variants in the ACADS gene and its relationship to the ethnic structure in Slovakia. BMC Med. Genet..

[6] Ju K., Bai F., Xu Y., Li Q., Su G., Jin Y., Chen H., Zhang S., Luan X. (2025). Structural Insights into Isovaleryl-Coenzyme A Dehydrogenase: Mechanisms of Substrate Specificity and Implications of Isovaleric Acidemia-Associated Mutations. Research.

[7] Wang B., Zhang Q., Gao A., Wang Q., Ma J., Li H., Wang T. (2019). New Ratios for Performance Improvement for Identifying Acyl-CoA Dehydrogenase Deficiencies in Expanded Newborn Screening: A Retrospective Study. Front. Genet..

[8] Naito E., Indo Y., Tanaka K. (1990). Identification of two variant short chain acyl-coenzyme A dehydrogenase alleles, each containing a different point mutation in a patient with short chain acyl-coenzyme A dehydrogenase deficiency. J. Clin. Investig..

[9] Battaile K.P., Mohsen A.W., Vockley J. (1996). Functional role of the active site glutamate-368 in rat short chain acyl-CoA dehydrogenase. Biochemistry.

[10] Hu H., Ma Q., Li W., Wang Y., Song W., Huang Y. (2024). Prevalence and Mutation Analysis of Short-Chain acyl-CoA Dehydrogenase Deficiency Detected by Newborn Screening in Hefei, China. Int. J. Neonatal Screen..

[11] Nochi Z., Olsen R.K.J., Gregersen N. (2017). Short-chain acyl-CoA dehydrogenase deficiency: From gene to cell pathology and possible disease mechanisms. J. Inherit. Metab. Dis..

[12] Pena L., Angle B., Burton B., Charrow J. (2012). Follow-up of patients with short-chain acyl-CoA dehydrogenase and isobutyryl-CoA dehydrogenase deficiencies identified through newborn screening: One center’s experience. Genet. Med..

[13] Kim Y.M., Cheon C.K., Park K.H., Park S., Kim G.H., Yoo H.W., Lee K.A., Ko J.M. (2016). Novel and Recurrent ACADS Mutations and Clinical Manifestations Observed in Korean Patients with Short-chain Acyl-coenzyme a Dehydrogenase Deficiency. Ann. Clin. Lab. Sci..

[14] Tonin R., Caciotti A., Funghini S., Pasquini E., Mooney S.D., Cai B., Proncopio E., Donati M.A., Baronio F., Bettocchi I. (2016). Clinical relevance of short-chain acyl-CoA dehydrogenase (SCAD) deficiency: Exploring the role of new variants including the first SCAD-disease-causing allele carrying a synonymous mutation. BBA Clin..

[15] Yang J., Zhu H., Zhang T., Ding J. (2021). Structure, substrate specificity, and catalytic mechanism of human D-2-HGDH and insights into pathogenicity of disease-associated mutations. Cell Discov..

[16] Jin S., Chen X., Yang J., Ding J. (2023). Lactate dehydrogenase D is a general dehydrogenase for D-2-hydroxyacids and is associated with D-lactic acidosis. Nat. Commun..

[17] Zhang Z., Tringides M.L., Morgan C.E., Miyagi M., Mears J.A., Hoppel C.L., Yu E.W. (2023). High-Resolution Structural Proteomics of Mitochondria Using the ‘Build and Retrieve’ Methodology. Mol. Cell. Proteom..

[18] Battaile K.P., Molin-Case J., Paschke R., Wang M., Bennett D., Vockley J., Kim J.J. (2002). Crystal structure of rat short chain acyl-CoA dehydrogenase complexed with acetoacetyl-CoA: Comparison with other acyl-CoA dehydrogenases. J. Biol. Chem..

[19] Gregersen N., Winter V.S., Corydon M.J., Corydon T.J., Rinaldo P., Ribes A., Martinez G., Bennett M.J., Vianey-Saban C., Bhala A. (1998). Identification of four new mutations in the short-chain acyl-CoA dehydrogenase (SCAD) gene in two patients: One of the variant alleles, 511C-->T, is present at an unexpectedly high frequency in the general population, as was the case for 625G-->A, together conferring susceptibility to ethylmalonic aciduria. Hum. Mol. Genet..

[20] Corydon M.J., Vockley J., Rinaldo P., Rhead W.J., Kjeldsen M., Winter V., Riggs C., Babovic-Vuksanovic D., Smeitink J., De Jong J. (2001). Role of common gene variations in the molecular pathogenesis of short-chain acyl-CoA dehydrogenase deficiency. Pediatr. Res..

[21] Sharpe A.J., McKenzie M. (2018). Mitochondrial Fatty Acid Oxidation Disorders Associated with Short-Chain Enoyl-CoA Hydratase (ECHS1) Deficiency. Cells.

[22] Narayanan B., Xia C., McAndrew R., Shen A.L., Kim J.P. (2024). Structural basis for expanded substrate specificities of human long chain acyl-CoA dehydrogenase and related acyl-CoA dehydrogenases. Sci. Rep..

[23] Schmidt S.P., Corydon T.J., Pedersen C.B., Bross P., Gregersen N. (2010). Misfolding of short-chain acyl-CoA dehydrogenase leads to mitochondrial fission and oxidative stress. Mol. Genet. Metab..

[24] Schmidt S.P., Corydon T.J., Pedersen C.B., Vang S., Palmfeldt J., Stenbroen V., Wanders R.J., Ruiter J.P., Gregersen N. (2011). Toxic response caused by a misfolding variant of the mitochondrial protein short-chain acyl-CoA dehydrogenase. J. Inherit. Metab. Dis..

[25] Edhager A.V., Stenbroen V., Nielsen N.S., Bross P., Olsen R.K.J., Gregersen N., Palmfeldt J. (2014). Proteomic investigation of cultivated fibroblasts from patients with mitochondrial short-chain acyl-CoA dehydrogenase deficiency. Mol. Genet. Metab..

[26] Marchi S., Giorgi C., Suski J.M., Agnoletto C., Bononi A., Bonora M., De Marchi E., Missiroli S., Patergnani S., Poletti F. (2012). Mitochondria-ros crosstalk in the control of cell death and aging. J. Signal Transduct..

[27] Mironova E., Kvetnoy I., Balazovskaia S., Antonov V., Poyarkov S., Mazzoccoli G. (2026). Mitochondria and Aging: Redox Balance Modulation as a New Approach to the Development of Innovative Geroprotectors (Fundamental and Applied Aspects). Int. J. Mol. Sci..

[28] McAndrew R.P., Wang Y., Mohsen A.W., He M., Vockley J., Kim J.J. (2008). Structural basis for substrate fatty acyl chain specificity: Crystal structure of human very-long-chain acyl-CoA dehydrogenase. J. Biol. Chem..

[29] Breilyn M.S., Kenny E.E., Abul-Husn N.S. (2023). Diverse and unselected adults with clinically relevant ACADS variants lack evidence of metabolic disease. Mol. Genet. Metab..

[30] Eleutherio E.C.A., Silva Magalhães R.S., de Araújo Brasil A., Monteiro Neto J.R., de Holanda Paranhos L. (2021). SOD1, more than just an antioxidant. Arch. Biochem. Biophys..

[31] Fridovich I. (1997). Superoxide anion radical (O_2_^·−^), superoxide dismutases, and related matters. J. Biol. Chem..

[32] Ott M., Gogvadze V., Orrenius S., Zhivotovsky B. (2007). Mitochondria, oxidative stress and cell death. Apoptosis.

[33] Su G., Ju K., Xu Y., Jin Y., Chen L., Zhang S., Luan X. (2024). Structural and biochemical basis of methylmalonate semialdehyde dehydrogenase ALDH6A1. Med. Plus.

[34] Area-Navarro M., Pastor-Moreno A., Scholz E., Cerqueira A., Tirado-Herranz A., Marcilla M., Canals F., Juan M., Palacio J.R., Alvarez I. (2025). Specific Instability of HLA-A*03:01 Expression in HEK-293 Cells. Int. J. Mol. Sci..

[35] Liu X., Wu L., Deng G., Chen G., Li N., Chu X., Li D. (2013). Comparative studies of Acyl-CoA dehydrogenases for monomethyl branched chain substrates in amino acid metabolism. Bioorg. Chem..

[36] Zeng J., Li D. (2004). Expression and purification of His-tagged rat mitochondrial medium-chain acyl-CoA dehydrogenase wild-type and Arg256 mutant proteins. Protein Expr. Purif..

[37] Bai F., Fan C., Lin X., Wang H.Y., Wu B., Feng C.L., Zhou R., Wu Y.W., Tang W. (2023). Hemin protects UVB-induced skin damage through inhibiting keratinocytes apoptosis and reducing neutrophil infiltration. J. Photochem. Photobiol. B.

