# Supplementary material for: Structure and Substrate Specificity of Human Short-Chain Acyl-CoA Dehydrogenase and Insights into Pathogenicity of Disease-Associated Mutations
Source: Int J Mol Sci. 2026 Mar 14;27(6):2657. doi: 10.3390/ijms27062657 (PMC13027082; doi:10.3390/ijms27062657)
Supplement: Supplementary file 1 [file ijms-27-02657-s001.zip › ijms-4156476-supplementary.pdf]

Supplementary Materials:

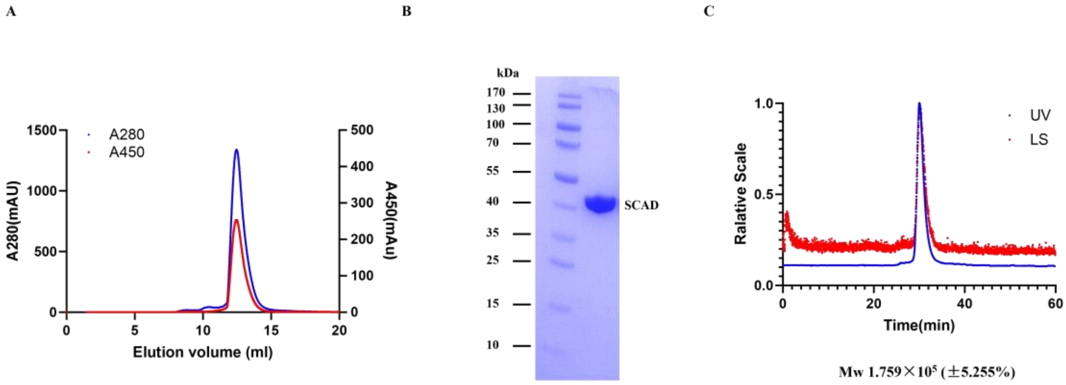

Figure S1. Purification of human SCAD. (**A**) Size-exclusion chromatography (SEC) analysis of SCAD. The ordinate represents the UV absorption value at 280 and 450 nm. Superdex 200 10/300 column (Cytiva) was used for gel filtration chromatography. (**B**) SDS-PAGE result of the purified SCAD. The elution peak fraction of SCAD from the SEC was analyzed by SDS-PAGE and stained with Coomassie blue. (**C**) Static light scattering (SLS) results for purified SCAD. The molecular weight of tetramer protein is 175 kDa.

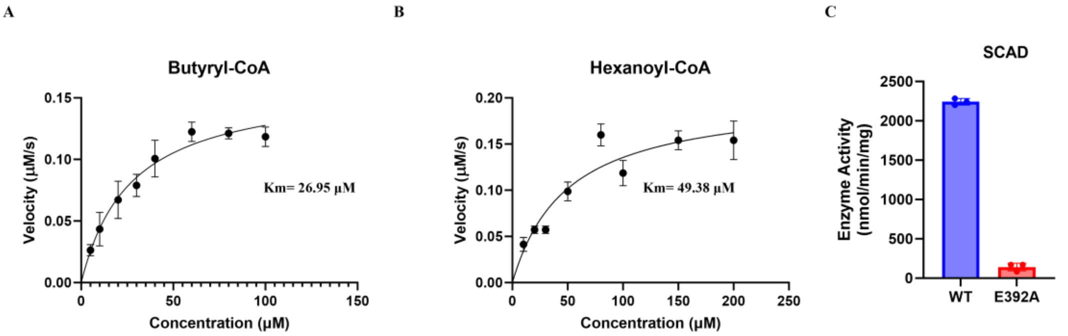

Figure S2. Enzymatic kinetic properties of SCAD. **(A-B)** The saturation curve of SCAD towards butyryl-CoA **(A)** and hexanoyl-CoA **(B)**. (**C)** The enzyme activities of wild-type SCAD and mutant E392A with 100 μM butyryl-CoA were shown in the figure. A standard assay mixture contained 20 mM phosphate buffer, pH 7.4, 100 μM DCPIP, 1.5 mM PMS, 30 μM EDTA, 20 μM FAD, and butyryl-CoA ranging from 1 to 100 μM. 0.75 μg of diluted protein was added to initiate the reaction with a final volume of 200 μl. Data points shown are the means of three independent determinations. The error bars represent the standard deviations.

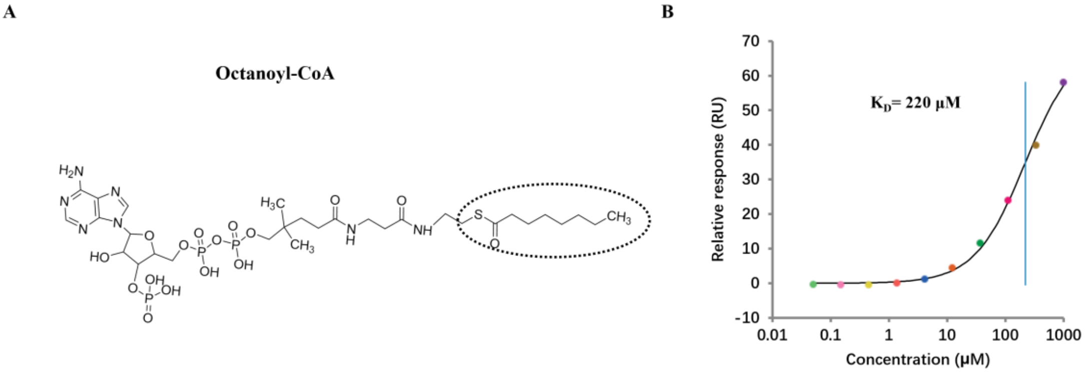

Figure S3. Affinity determination of SCAD for Octanoyl-CoA using surface plasmon resonance (SPR). (**A**) The chemical structures of Octanoyl-CoA. The acyl chain is highlighted by a circle. (**B**) SPR analysis of SCAD binding with hexanoyl-CoA. The solid line shows the global fit to the binding data (colored dots), yielding a KD of 220 μM, which is indicated by the vertical blue line.

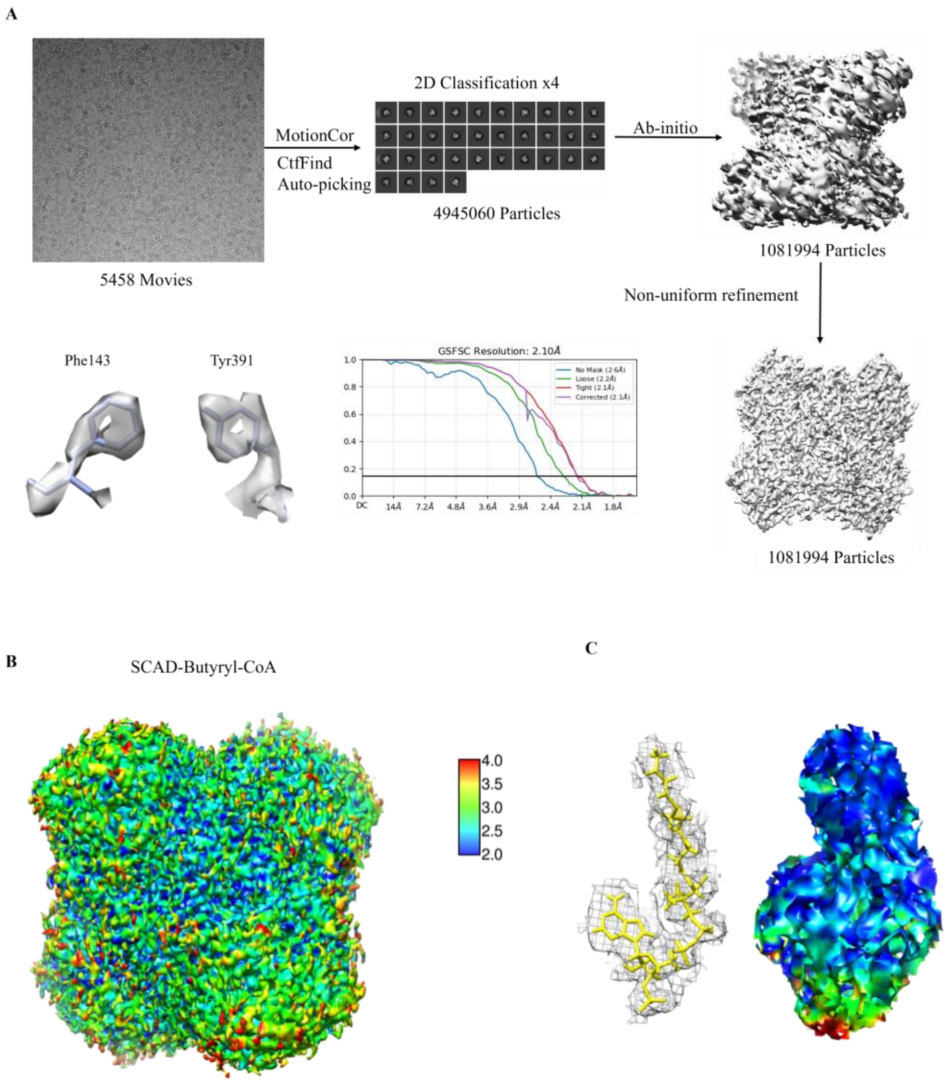

Figure S4. Cryo-EM data processing and validation of the SCAD-Butyryl-CoA. (**A**) Data processing workflows for the SCAD-Butyryl-CoA including data acquisition, processing, model building and refinement. The FSC curve indicates a global resolution of 2.10 Å. Representative densities for Phe143 and Tyr391 show clear side-chain features, verifying the map quality. (**B**) Local resolution maps for the SCAD-Butyryl-CoA. (**C**) Analysis of the binding pocket. Left: Butyryl-CoA (sticks) fitted into the experimental density (mesh). Right: Local resolution map of the binding pocket, confirming the high resolution of the ligand-binding core.

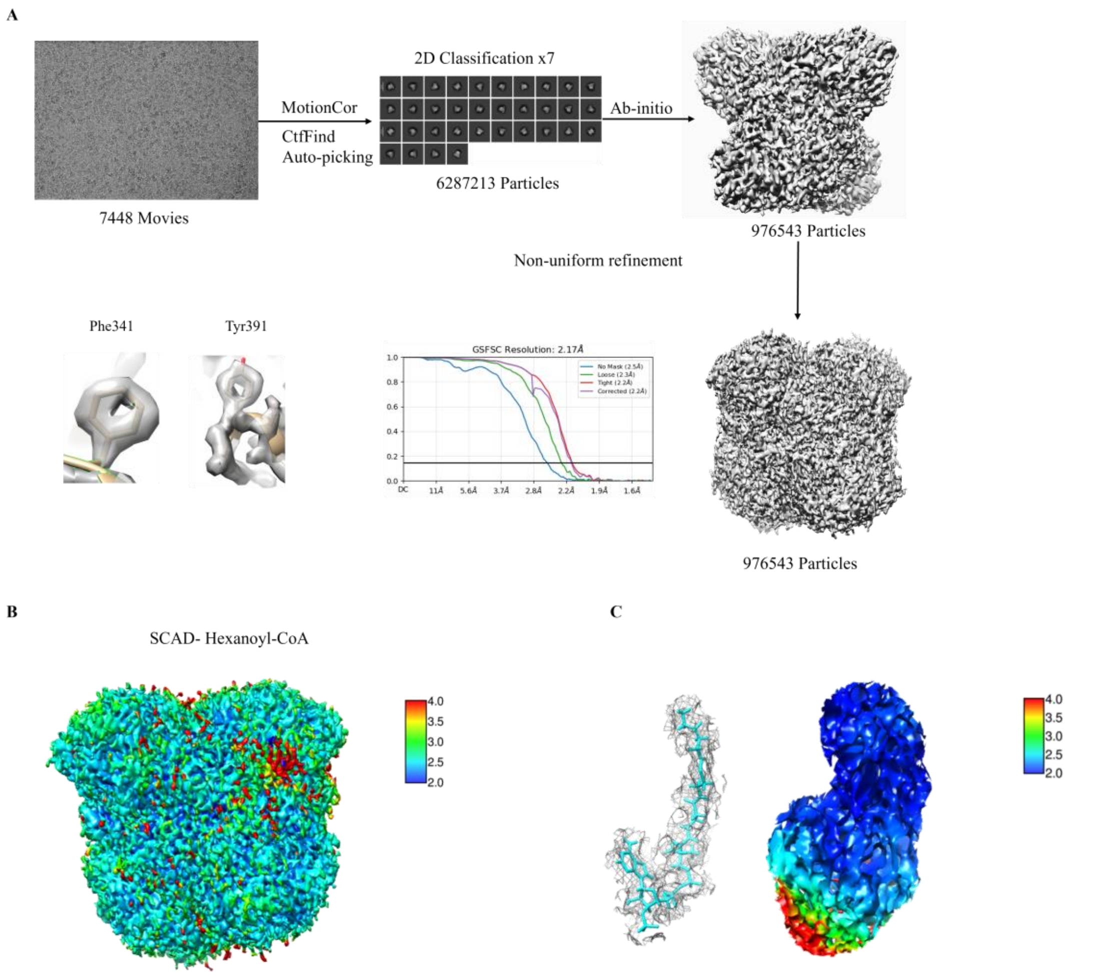

Figure S5. Cryo-EM data processing and validation of the SCAD-Hexanoyl-CoA. (**A**) Data processing workflows for the SCAD-Hexanoyl-CoA including data acquisition, processing, model building and refinement. The FSC curve indicates a global resolution of 2.17 Å. Representative densities for Phe341 and Tyr391 show clear side-chain features, verifying the map quality. (**B**) Local resolution maps of the SCAD-Hexanoyl-CoA. (**C**) Analysis of the binding pocket. Left: Hexanoyl-CoA (sticks) fitted into the experimental density (mesh). Right: Local resolution map of the binding pocket, confirming the high resolution of the ligand-binding core.

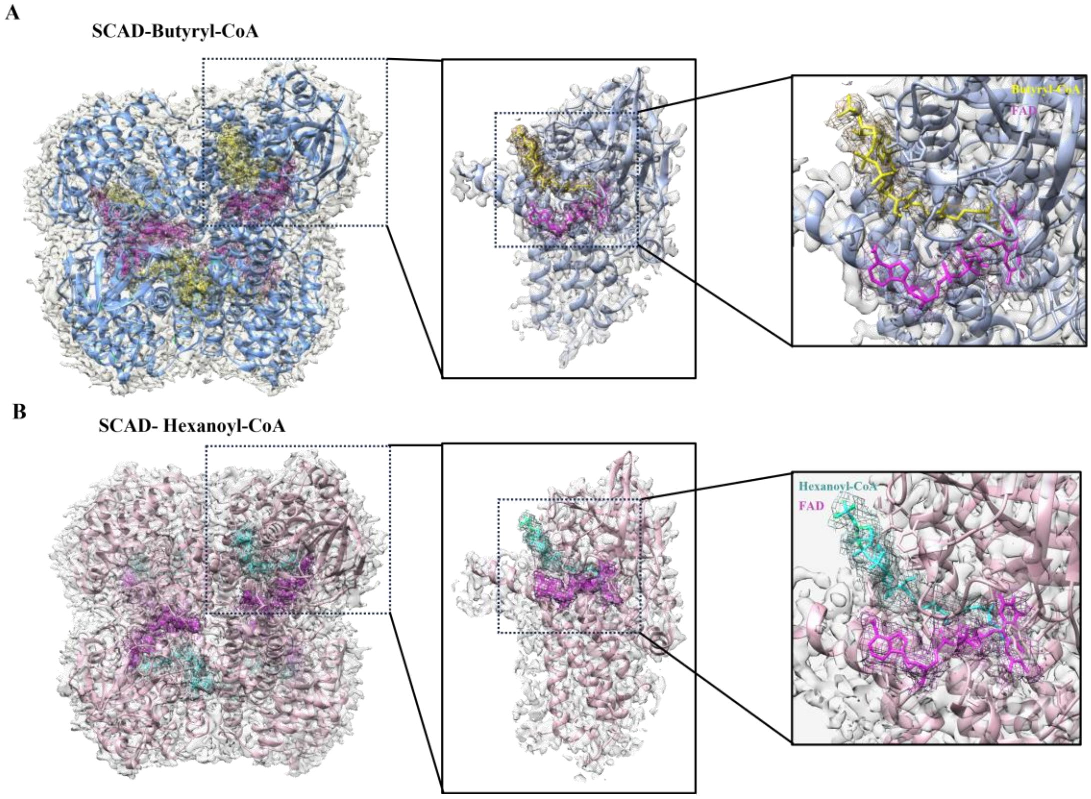

Figure S6. Cryo-EM density maps and atomic models of the SCAD complexes. (**A**) Cryo-EM map of the SCAD-Butyryl-CoA complex is displayed as a gray surface, overlaid with the atomic model (blue ribbons). The density corresponding to the butyryl-CoA is highlighted in yellow, with the FAD density highlighted in violet. Close-up views show the atomic models of Butyryl-CoA (yellow) and FAD (violet) fitted into the cryo-EM density (mesh). (**B**) Similar representation for the SCAD-Hexanoyl-CoA complex (pink ribbons), with the Hexanoyl-CoA density highlighted in cyan. Detailed densities for Hexanoyl-CoA and FAD are shown in the insets.

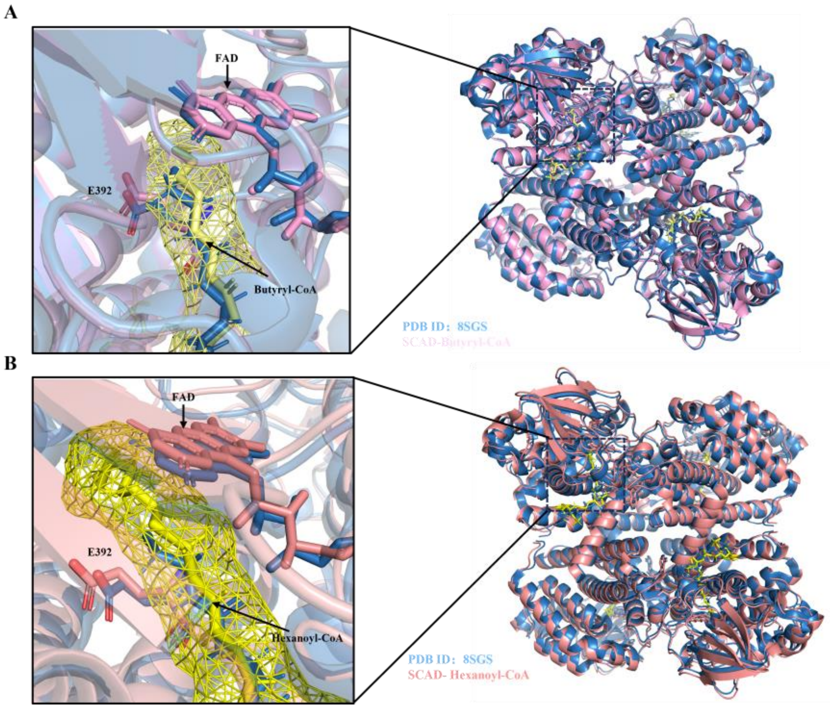

Figure S7. Structural comparison of the SCAD complexes with the previous structure (PDB ID: 8SGS). (**A**) Alignment of the SCAD-Butyryl-CoA complex (pink) with the 8SGS structure (blue). RMSD is 0.5 Å with 1486 aligned atoms. Zoom-in view of the substrate-binding pocket is displayed on the left. (**B**) Alignment of the SCAD-Hexanoyl-CoA complex (salmon) with the 8SGS structure (blue). RMSD is 1.1 Å with 1518 aligned atoms. Butyryl-CoA / Hexanoyl-CoA is shown as yellow sticks.

Supplementary **Table S1**. Potential functional roles of the disease-associated mutations of SCAD

Constructs	Possible impact	Purified protein yield (mg/L)
WT	/	1.917
R46W	Protein folding and stability	NA
P55L	Protein folding and stability	0.055
R107C	Protein folding and stability	0.030
L154R	FAD binding	0.044
G160S	FAD binding	0.135
S161G	Substrate binding	0.579
W177R	Protein folding and stability	NA
R272C	Substrate binding	0.120
K313E	Protein folding and stability	0.015
R325W	Protein folding and stability	NA
R330C	Protein folding and stability	NA
R330H	Protein folding and stability	0.244
E344G	Protein folding and stability	NA
S353L	Protein folding and stability	NA
Q365H	Protein folding and stability	NA
G371V	Protein folding and stability	0.191
L400V	Substrate binding	0.953
R380W	Protein folding and stability	NA
R383C	Protein folding and stability	NA

Supplementary **Table S2.** Cryo-EM data collection, processing, model refinement and validation parameters

	SCAD-Bu	SCAD-He
**Data Collection**		
EM equipment	TFS Titan Krios	TFS Titan Krios
Voltage (kV)	300	300
Detector	Gatan K2 Summit	TFS Falcon 4
Magnification	165,000	165,000
Pixel size (Å)	0.82	0.729
Electron dose (e^-/$Å^2$)	40	40
Defocus range (μm)	-0.8~-1.2	-0.8~-1.2
**Reconstruction**		
Software	CryoSPARC 4.7.1	CryoSPARC 4.7.1
Number of used Particles	1,081,994	976,543
Symmetry	C1	C1
Final Resolution (Å)	2.1	2.17
**Model building**		
Software	CooT 0.9.8.96	CooT 0.9.8.96
**Refinement**		
Software	PHENIX	PHENIX
**Validation**		
B-factor ($Å^2$)		
Protein (min/max/mean)	6.28/71.29/23.61	1.91/95.23/26.99
Bond angles (°)	0.578	0.534
MolProbity Score	1.59	1.41
All-Atom Clash score	6.53	4.42
Ramachandran plot statistics (%)		
Preferred (%)	98.48	98.61
Allowed (%)	1.52	1.39
Disallowed (%)	0	0
**Data availability**		
EMDB entry	EMD-66500	EMD-66565
PDB entry	9X3I	9X4S
